# Isolation of Radial Glia-Like Neural Stem Cells from Fetal and Adult Mouse Forebrain via Selective Adhesion to a Novel Adhesive Peptide-Conjugate

**DOI:** 10.1371/journal.pone.0028538

**Published:** 2011-12-02

**Authors:** Károly Markó, Tímea Kőhidi, Nóra Hádinger, Márta Jelitai, Gábor Mező, Emília Madarász

**Affiliations:** 1 Institute of Experimental Medicine of Hungarian Academy of Sciences, Budapest, Hungary; 2 Research Group of Peptide Chemistry, Hungarian Academy of Sciences, Eötvös Loránd University, Budapest, Hungary; Biological Research Center of the Hungarian Academy of Sciences, Hungary

## Abstract

Preferential adhesion of neural stem cells to surfaces covered with a novel synthetic adhesive polypeptide (AK-cyclo[RGDfC]) provided a unique, rapid procedure for isolating radial glia-like cells from both fetal and adult rodent brain. Radial glia-like (RGl) neural stem/progenitor cells grew readily on the peptide-covered surfaces under serum-free culture conditions in the presence of EGF as the only growth factor supplement. Proliferating cells derived either from fetal (E 14.5) forebrain or from different regions of the adult brain maintained several radial glia-specific features including nestin, RC2 immunoreactivity and *Pax6, Sox2, Blbp, Glast* gene expression. Proliferating RGl cells were obtained also from non-neurogenic zones including the parenchyma of the adult cerebral cortex and dorsal midbrain. Continuous proliferation allowed isolating one-cell derived clones of radial glia-like cells. All clones generated neurons, astrocytes and oligodendrocytes under appropriate inducing conditions. Electrophysiological characterization indicated that passive conductance with large delayed rectifying potassium current might be a uniform feature of non-induced radial glia-like cells. Upon induction, all clones gave rise to GABAergic neurons. Significant differences were found, however, among the clones in the generation of glutamatergic and cathecolamine-synthesizing neurons and in the production of oligodendrocytes.

## Introduction

Proliferating cells with potential to generate more than one neural cell types can be isolated from the mammalian CNS at any ages [1]. Diverse cell populations corresponding to the criteria of “neural stemness” (e.g. self-renewal, ability to generate committed neural progenies) exist in the entire lifespan of mammals starting from the early embryonic neural plate [2] up to the neurogenic regions of the adult brain [3], [4]. Beside resident stem cells in the adult neurogenic zones, the subventricular zone (SVZ) of the lateral ventricles and the subgranular zone (SGZ) of the hippocampus, quiescent and active progenitor cells seem to persist in the brain parenchyma [5], as well. The diverse neural stem/progenitor populations should be characterized, but for this end, purified cell preparations are required with preserved native features.

Embryonic radial glial cells representing the neurogenic population in the embryonic neural tissue [2] expand through distinct layers of the developing neural tube and brain vesicles. Their apical and basal parts are settled in the laminin-rich ventricular and pial zones. Large areas of the cell surfaces, however, span through the intermedier zone where fibronectin is the predominant extracellular matrix molecule [6]. Fibronectin and a number of other ECM molecules bind to different integrin receptors with different affinities. Stimulated integrin receptors, besides mediating adhesion, initiate intracellular responses supporting cell-survival, proliferation and/or differentiation [7], [8].

According to previous results [9], non-differentiated progenitor-like cells can be separated from mature neurons and macroglia by adhesive preferences. We found that a cyclic pentapeptide (cyclo[RGDfC]) containing a rigid RGD sequence, selectively interferes with the adhesion and survival of non-differentiated cells, among them cloned NE-4C [10] neurepithelial stem cells.

The cyclic RGD motif is a high-affinity ligand of α_v_β_3_/α_v_β_5_ type integrins [11], those binding preferentially vitronectin and fibronectin. These integrins were suggested to play important roles in radial glia functions, including the guidance of neuronal migration [12] and vasculogenesis [13]. In the developing brain,α_v_ [14] and β_3_ [15] integrin subunits are carried predominantly by radial glial cells.

By conjugating the cyclo[RGDfC] motif to a branching polypeptide backbone [16], a novel brush-like cell-adhesive molecule, AK-cyclo[RGDfC], was obtained [9], where the integrin-ligand RGD sequence is embedded in a cyclic pentapeptide (c[RGDfC]), and the ring is bound to the N-termini of D/L-alanine side-chains hanging from a poly-L-lysine backbone.

Radial glia-like neural stem/progenitor cells adhered rapidly to AK-cyclo[RGDfC]-coated surfaces in serum-free culture conditions. Adhesion-based selection and serum-free propagation allowed growing and cloning radial glia-like (RGl) cells from both, fetal forebrain and various adult brain regions.

Here we present methods for isolation, propagation and in vitro differentiation of RGl cells, and give a summary on molecular, physiological and developmental characteristics of different RGl clones. The data demonstrate that i) appropriate adhesive conditions allow isolating, long-term culturing and characterising radial glia-like cells in chemically defined, xeno-free cultures, and ii) AK-cyclo[RGDfC]-adherent cells with radial glia-like features can be isolated from fairly different regions of the adult mouse brain.

## Results

### Stem/progenitor cells from the fetal mouse forebrain

**Figure 1:**
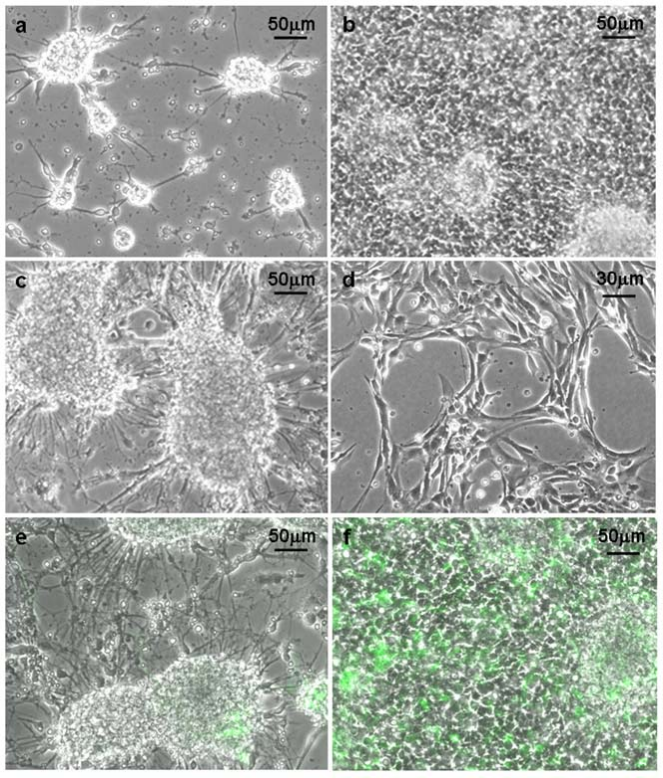
Neural cultures plated onto PLL- or AK-cyclo[RGDfC]- coated surfaces. Phase-contrast view of primary cultures of fetal (E14.5) mouse forebrain cells on AK-cyclo[RGDfC]-coated surface, on the 2^nd^ (**a**) and 6^th^ (**b**) days after plating, and on poly-L-lysine (**c**) coated surface on the 6^th^ day after plating. On AK-cyclo[RGDfC] morphologically homogeneous cultures of radial glia-like cells developed after the first passage (**d**). In primary cultures prepared from the forebrain of hGFAP-GFP mouse embryos (E14.5), GFP-expressing cells colonized the AK-cyclo[RGDfC] surface (**f**), while stayed inside the aggregates on PLL (**e**) (6^th^ day after plating).

On the first 2-3 days after seeding, the primary cultures of fetal neural cells showed the usual clustered morphology (Figure 1a) on both, PLL (poly-L-lysine; a commonly used adhesive polypeptide) and AK-cyclo[RGDfC] substrates. By the end of the first week however, dense population of surface-attached cells developed on the AK-cyclo[RGDfC]-coated surfaces (Figure 1b), while very few substrate-attached cells were seen on PLL substrate, instead, the cultures were dominated by neuronal aggregates interconnected by bundles of neurites (Figure 1c).

Primary neural cultures were prepared as above also from hGFAP-GFP transgenic [17] mouse embryos. In this transgenic strain, the human GFAP-promoter is active well before the formation of astrocytes, and the green fluorescent protein is expressed by radial glia-like stem/progenitor cells [18]. From cell suspensions prepared from E14.5 hGFAP-GFP mouse forebrains, GFP-positive cells colonized readily the AK-cyclo[RGDfC] coated surfaces (Figure 1f), while hardly attached to the PLL-coat (Figure 1e), indicating that neural stem/progenitor cells adhered preferentially to AK-cyclo[RGDfC].

**Figure 2:**
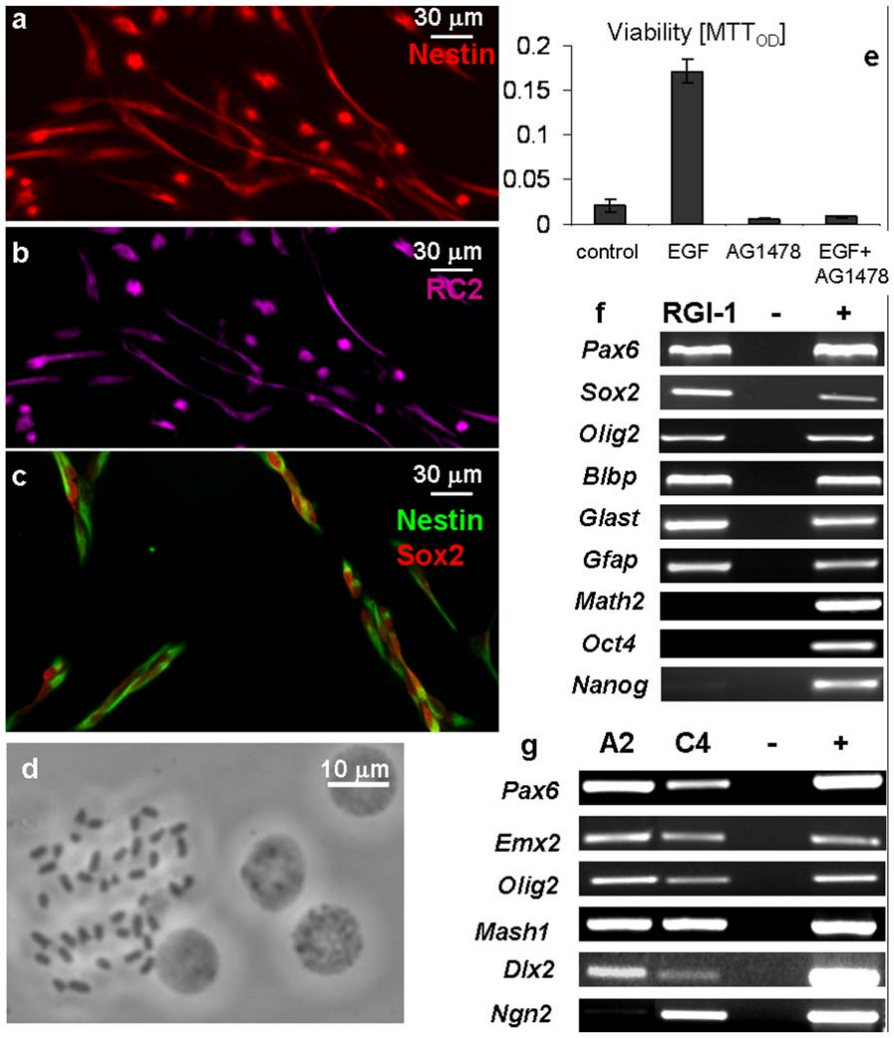
Characteristics of fetal radial glia-like cell clones. Cultured radial glia-like cells display nestin- (**a, c**) RC2- (**b**) and Sox2-immunoreactivity (**c**). Cloned radial glia-like (RGl-1) cells contain euploid number (n = 40) of chromosomes (**d**). Cell viability was determined by MTT-assay in cultures maintained with EGF (20 ng/ml), with the EGF receptor antagonist AG 1478 (10^−7^ M) or with both (**e**). Averages and standard deviations were calculated from 6-8 identically treated sister-cultures; OD: optical density. Radial glia- and/or neural stem cell-specific genes were active in cloned RGl-1 cells, while “pluripotency markers” (*Oct4, Nanog*) and the neuron-specific gene (*Math2*) were not transcribed (**f**). *GFAP* was present at the mRNA-level, but the protein could not be detected. From the investigated region-specific genes, only *Ngn2* showed alteration between RGl-clones derived from the ventral (RGl-GFP-A2) and dorsal (RGl-GFP-C4) regions of the embryonic (E14.5) forebrain (**g**).

The cells adhering to AK-cyclo[RGDfC] spread and proliferated in complete RGl-medium, e.g. without serum and in the presence of EGF and insulin (present in B27 supplement) as only external growth factor-compounds. After a week, the cultures were split and the cells were transferred to fresh AK-cyclo[RGDfC]-coated dishes. In over twenty independent series of experiments, the first passage resulted in apparently homogenous cultures of elongated, proliferating cells (Figure 1d). The cellular composition of the cultures was checked also by immunocytochemical staining along more then 10 passages. The proportion of neurons and astrocytes (identified by III β-tubulin- or GFAP-immunreactivities, respectively) decreased drastically with consecutive passages and became negligible (<0.1%) after 3 passages. The vast majority of cells displayed nestin- (Figure 2a,c), RC2- (Figure 2b), Sox2- (Figure 2c) immunoreactivity, indicating a radial glia-like, neural stem/progenitor phenotype. After 3 or 4 passages, the cell composition and morphology of the cultures were stabilized. The growth rate or the expression of investigated genes did not change with further (more than 10) passages. The cultures could be maintained as adherent monolayers of RC2-immunopositive, elongated cells. Overgrowth-induced cell death or spontaneous differentiation in subconfluent cultures was not detected.

The continuous proliferation of non-confluent cells allowed separating one-cell derived RGl clones. Several clones were established from CD1- and CD1/EGFP mice [19], and a few of them (RGl-1, RGl-GFP-A2 and RGl-GFP-C4) were characterized in details (Table 1). After more than 20 passages, cells of all RGl-clones contained 40 chromosomes (Figure 2d) indicating a steady euploidity and genetic stability in spite of rapid *in vitro* propagation.

**Table 1:** Radial glia-like cell clones.

Clone	Embryonic (E14.5)			Adult (P 50-75)						
	A2	C4	RGl-1	HC_A	CTX_H	MES_D	SVZ_I	SVZ_K	SVZ_T	SVZ_M
Source tissue	ventral forebrain	dorsal forebrain	total forebrain	hippocampus	parietal cortex	dorsolateral colliculus sup.	SVZ	SVZ	SVZ	SVZ
RC2/Nestin immunreactivity	+	+	+	+	+	+	+	+	+	+
GFAP immunreactivity	-	-	-	+	+	+	+	+	+	+
Gene expression										
Sox2	+	+	+	+	+	+	+	+	+	+
Olig2	+	+	ni	+	+	+	+	+	+	+
Pax6	+	+	+	+	+	+	+	+	ni	ni
blbp	+	+	+	+	+	+	+	+	+	+
Glast	+	+	+	+	+	+	+	+	+	+
Oct4	-	-	-	+?	-	-	-	-	-	-
Emx2	+	+	+	+	+	+	+	+	+	+
Nkx2.1	-	-	-	-	-	-	-	-	-	-
Gbx2	ni	ni	+	+	+	+	+	+	+	+
Dlx2	+	+	+	+	+	+	+	+	ni	ni
Hoxb2	ni	ni	-	-	-	-	-	-	ni	ni
Ngn2	-	+	+	+	+	+	+	-	+	+
Mash1	+	+	+	+	+	+	+	+	+	+
GFAP	+	+	+	+	+	+	+	+	+	+
Math2	-	-	ni	-	-	+	-	-	-	-

RGl cells displayed severe EGF-dependence. In low density cultures of RGl-1 cells, withdrawal of EGF from the medium or blocking EGF signalling by AG1478 (0.25 µM; Calbiochem) resulted in complete death of cells (Figure 2e).

The mRNA profile of all embryo-derived RGl clones revealed the characteristics of radial glia-like neural stem cells. They expressed *Pax6*, *Sox2*, *Olig2*, *Glast* and *Blbp*, while from pluripotency genes, *Nanog* mRNA was detected occasionally at low level and *Oct4* was not transcribed (Figure 2f). Interestingly, GFAP mRNAs was also produced but the protein could not be detected with anti-GFAP antibodies.

Many of the investigated positional genes (*Pax6, Olig2, Dlx2, Emx2*) characterizing regional determination in the developing forebrain, were expressed by all clones, regardless of their dorsal (pallial; RGl-GFP-C4), or ventral (subpallial; RGl-GFP-A2) origin (Figure 2g). From the proneural genes [20], *Ngn2* was expressed at high level in clones of dorsal origin, thus recapitulating in vivo expression while *Mash1* was expressed by all investigated clones regardless of their origin.

### Radial glia-like neural progenitors isolated from the adult mouse brain

**Figure 3:**
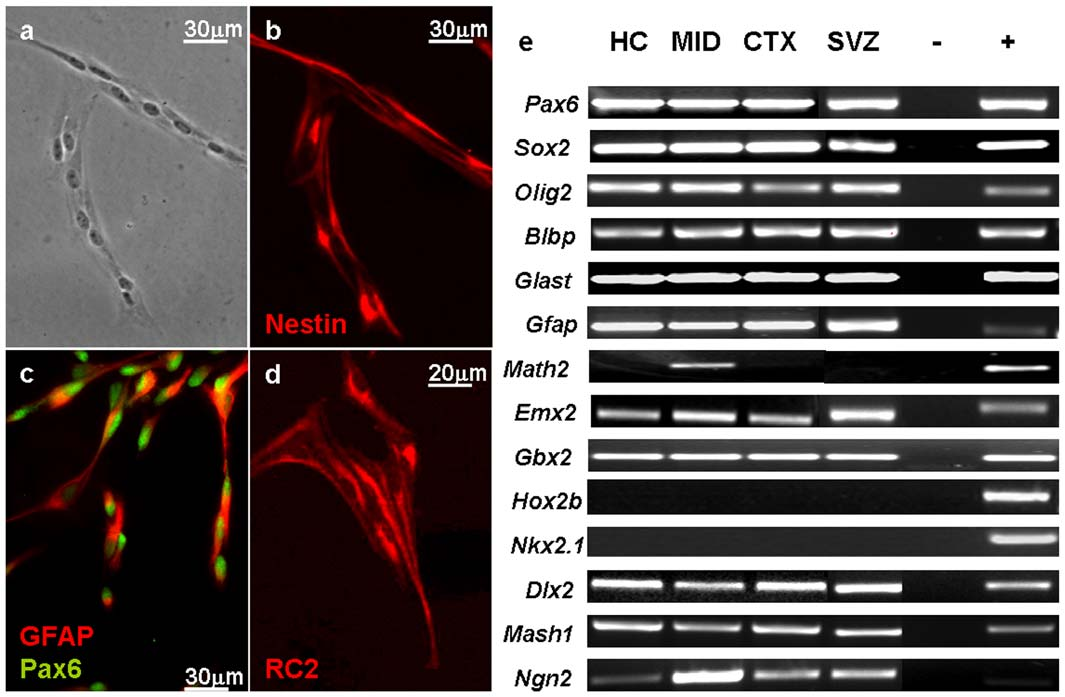
Characteristics of radial glia-like cell clones derived from adult mouse brain. Adult brain-derived radial glia-like cells (after the first passage) showed elongated cell shape (**a**), nestin- (**b**) RC2- (**d**) and Pax6- (**c**) immunoreactivity, like those derived from fetal forebrains, but in contrast to embryonic clones, they displayed GFAP-immunoreactivity (**c**). Cells of adult-derived RGl clones expressed genes characteristic to radial glial cells and many of the investigated positional genes (**e**).

Cell suspensions were prepared from both, neurogenic regions (the subependymal zone of the lateral ventricles and the hippocampus) and non-neurogenic (cortex and midbrain) parenchyma of the adult (P50-74) mouse brain. From the adult-derived cell suspensions, only very few cells (<1‰) attached to the AK-cyclo[RGDfC] adhesive coats. The attached cells, however, produced expanding colonies in the EGF supplemented defined medium, within three days. The rapidly proliferating cells displayed spindle-like morphology, and expressed radial glia/neural stem cell markers (Figure 3b, 3c, 3d). In the presence of EGF, all adult brain-derived radial glia-like cells produced high-density monolayer cultures in a 10-day period. Subsequent passages resulted in homogeneous cultures of non-differentiated, proliferating cells except the midbrain-derived cultures, where neurons were spontaneously formed under the same conditions. In contrast to the fetal RGl cells, adult-derived cells contained GFAP mRNA at high level and displayed GFAP-immunopositivity (Figure 3a, 3c, 3e).

Several one-cell-derived clones were established from distinct brain regions including the forebrain subependymal zone, hippocampus, cortex and dorsal midbrain (Table 1). All clones expressed radial glial marker genes (Figure 3e) and preserved euploidity (chromosome number 2n = 40). Similarly to embryo-derived RGl-clones, all adult-derived clones expressed “positional” genes (as *Dlx2, Emx2, Pax6*, *Gbx2*, *Ngn2, Mash1*) which, during *in vivo* development, are not transcribed in overlapping territories (Figure 3e, Table 1). On the other hand, the hindbrain/spinal cord marker Hoxb2, and more surprisingly, the ventral forebrain marker Nkx2.1 were not expressed by any of the clones.

### Electrophysiological properties of radial glia-like cells

**Figure 4:**
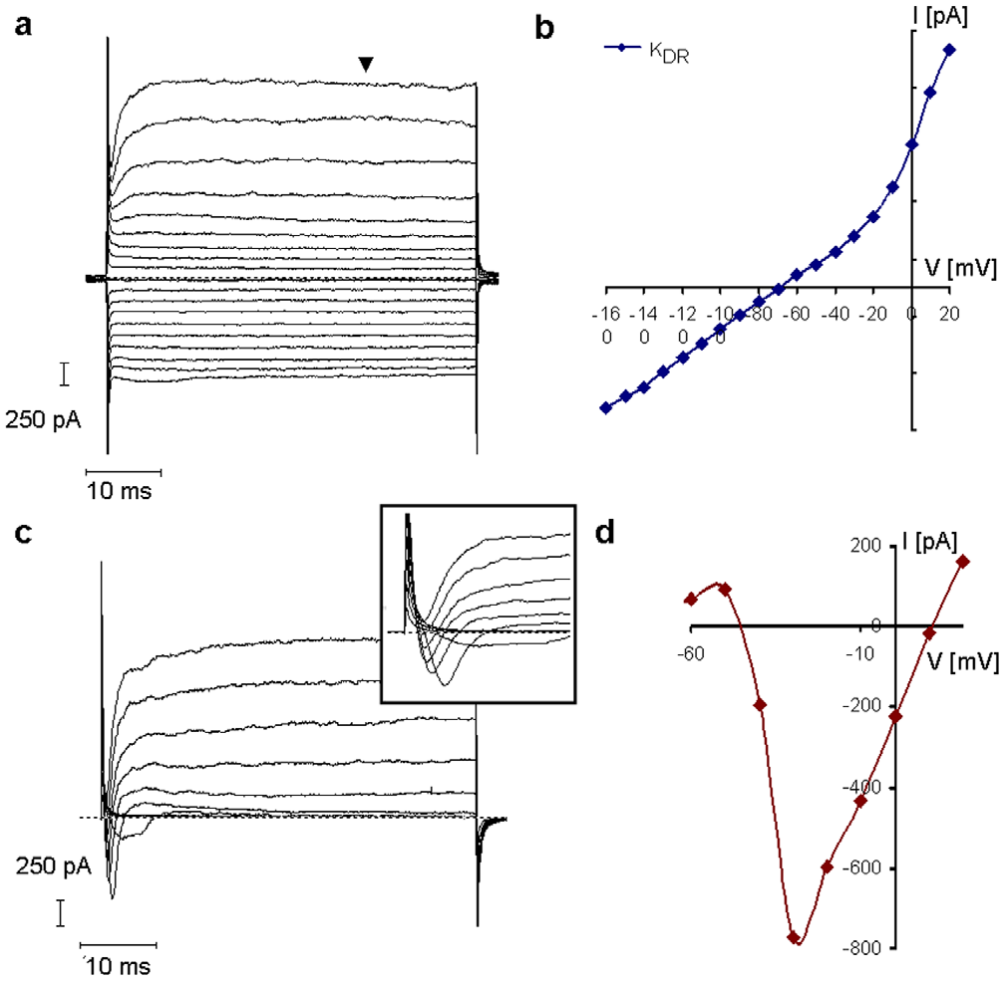
Electrophysiological characteristics of radial glia-like cells. Electrophysiological characteristics of cloned RGl-1 cells (**a**, **b**) and RGl-1 derived neurons (**c**, **d**) were detected by whole-cell patch-clamp recording. Large passive conductance together with K_DR_ current (**a**) and current/voltage (I-V) relationship (**b**) are shown from a representative RGl-1 cell. Voltage-dependent inward Na-currents with small amplitude (**c**) were detected from primitive, differentiating RGl-derived neurons (n = 8). A representative current-profile and its current/voltage (I-V) relationship (**d**) are shown. The current traces were obtained by clamping the cell membrane from a -70 mV holding potential to values ranging from -160 mV to +20 mV, at 10 mV intervals.

Radial glia-like cells displayed large passive conductance (4.2±0.7 nS) (Figure 4), regardless of fetal or adult origin. Beside the time- and voltage independent passive currents, RGl cells showed voltage-dependent outward potassium currents with a threshold of -30mV - -40 mV (Figure 4b). The outward potassium currents were not inactivated within the 50 ms duration of test pulses (Figure 4a) representing the features of delayed rectifying potassium currents (K_DR_). In non-differentiated RGl cells, inward rectifying potassium current or voltage dependent currents other than K_DR_ were not detected. While the peak amplitude of K_DR_ was 780±80 pA in developing neurons, radial glia-like cells displayed K_DR_ currents with amplitudes of 1781±238 pA, at 20 mV holding potential (Figure 4b). The passive conductance with large delayed rectifying potassium current seemed to be a characteristic of non-induced radial glia-like cells.

### Inducibility of neural tissue-type differentiation of cloned RGl cells

**Figure 5:**
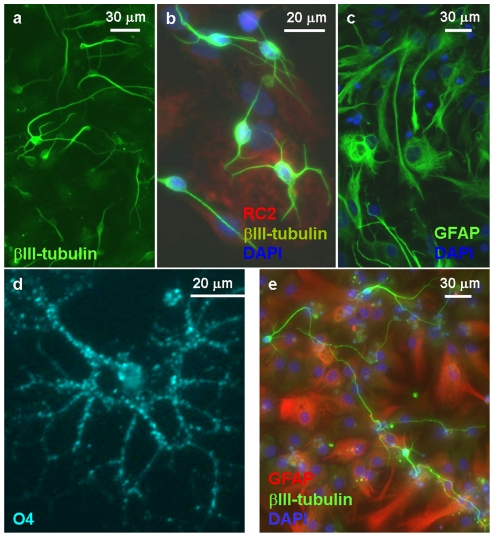
Neural differentiation of radial glia-like cells. Withdrawal of EGF resulted in neuron formation in a 6-day period in both, embryo- (**a**, **b**) and adult- (**e**) derived RGl cell cultures. βIII-tubulin-positive neurons appeared on the top of flat substrate-attached cells which were RC2-positive and GFAP-negative in embryo-derived cultures (**b**), but displayed GFAP-immunoreactivity in adult-derived cultures (**e**). GFAP-positive astrocytes appeared in the cultures of embryo-derived RGl cells only in response to supplementation with FCS (**c**). A 4+4-day induction period (Glaser et al, 2007) evoked the appearance of O4-immunopositive oligodendrocyte-precursors in each investigated RGl clones (**d**).

**Figure 6:**
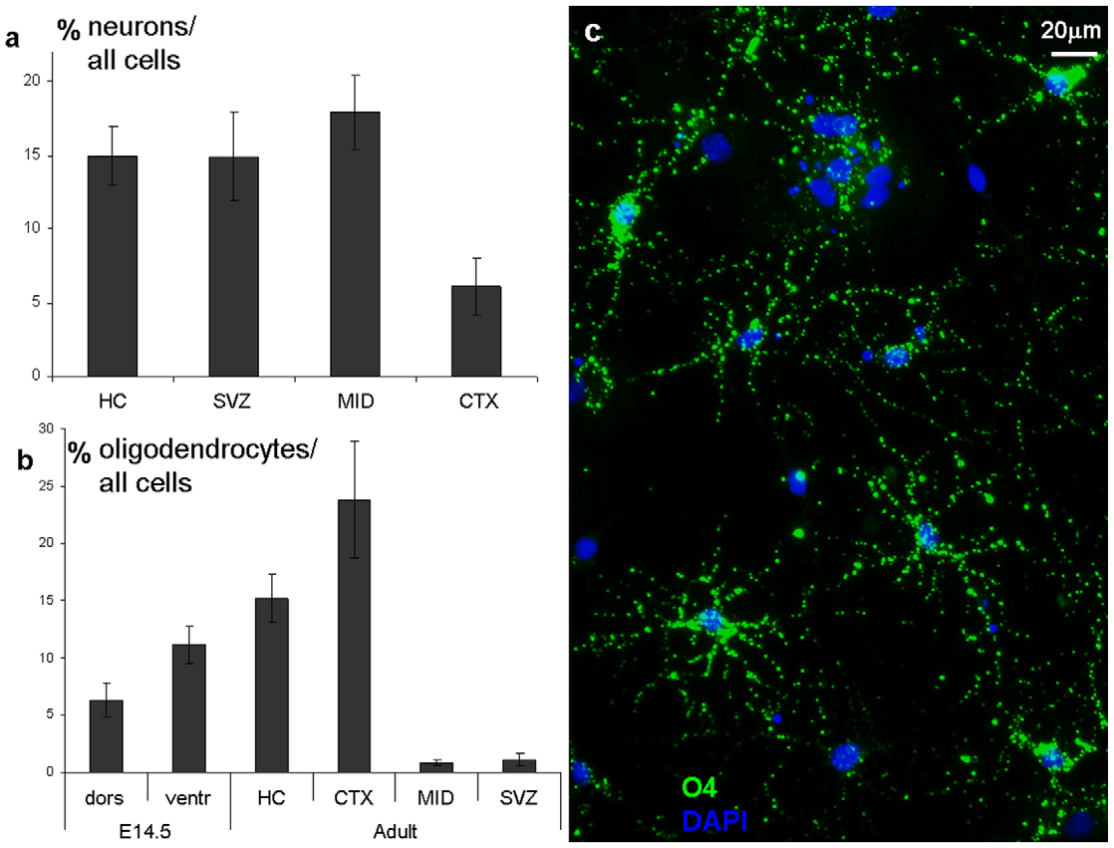
Rate of neuron- and oligodendrocyte production. The rate of neuron and oligodendrocyte production by adult-derived clones showed marked differences. After six days of EGF-withdrawal, adult cortex-derived RGl cells (CTX) gave rise to significantly less neurons than any other clones (**a**). RGl cells derived from the fetal ventral forebrain (clone A2) produced almost twofold more oligodendrocytes than those of dorsal origin (clone C4) (**b**). Adult RGl cells with hippocampal (HC; clone HC_A) and cortical (CTX; clone CTX_H) origin generated significant amount of oligodendrocytes (O4-immunopositive cells; **c**), while those of SVZ- (clone: SVZ_M) and midbrain (MID; clone MES_D)-origin produced significantly less O4-positive cells (∼1% of total cells).

Withdrawal of EGF from dense confluent cultures lead to the appearance of differentiating neurons (Figure 5a, 5b, 5e) (in contrast to low density cultures, where EGF withdrawal caused cell death). Process-bearing, βIII-tubulin-immunoreactive neurons (Figure 5a, 5b, 5e) appeared on the top of a monolayer of flat, substrate-attached cells in a 5–7 day period after EGF-withdrawal in all clones, regardless of their origin. Treatment with all-trans retinoic acid (10^−8^–10^−6^ M; known to induce neural differentiation of embryonic/early neural stem cells), on the other hand, did not induce neural differentiation either in embryo- or adult-derived RGl clones. While neurons were produced by all RGl clones, the rate of neuron formation varied significantly. Besides fetal RGl cells, clones isolated from the adult SVZ, hippocampus and rather surprisingly, from the adult midbrain gave rise to high amount of neurons, while the investigated adult cortical clones produced much less nerve cells (Figure 6a).

Cells differentiating upon EGF withdrawal showed high input resistance and consequently, low passive conductance (0.6±0.08 nS). Delayed rectifying potassium currents were recorded from both non-induced RGl cells and differentiating neuronal precursors or neurons, but with different amplitudes (Figure 4c). In process-bearing cells with neuronal morphology, voltage-dependent inward sodium currents were detected besides K_DR_ currents, indicating the advancement of physiological differentiation (Figure 4c, 4d).

In embryo-derived RGl cultures, withdrawal of EGF did not initiate the formation of GFAP-immunoreactive cells. In these cultures, differentiating neurons resided on the top of RC2-immunpositive substrate-attached cells (Figure 5b). GFAP-positive astrocytes, however, were rapidly formed (in about 72 hours) if the culture medium was supplemented with 5% FCS (Figure 5c). In contrast to fetal RGl cultures, substrate-attached cells displayed GFAP-immunoreactivity (Figure 5e) in all adult-derived clones with or without induction. Withdrawal of EGF from dense adult-derived RGl cultures, however, resulted in flattening of originally spindle-shaped substrate-attached cells and acquiring astrocyte-like morphology.

Generation of oligodendrocytes (Figure 5d, 6c) could be provoked in all RGl clones by the two-step differentiation protocol described by Glaser et al [21]. In an 8-day period, O4-immunopositive premature oligodendrocytes developed in all clones, but with different frequency (Figure 6b). The proportion of oligodendrocyte-precursors was around 6% and 12% in differentiated cultures of fetal dorsal and ventral forebrain-derived RGl cells, respectively. The highest (23.8±5.05%) oligodendrocyte-precursor density was produced by adult cortical (CTX) and hippocampal (HC) RGl cells, while midbrain (MID) and SVZ-derived RGl clones generated significantly less (around 1%) oligodendrocyte precursors (Figure 6b).

### Cloned RGl cells give rise to neurons with diverse neurotransmitter phenotypes

**Figure 7:**
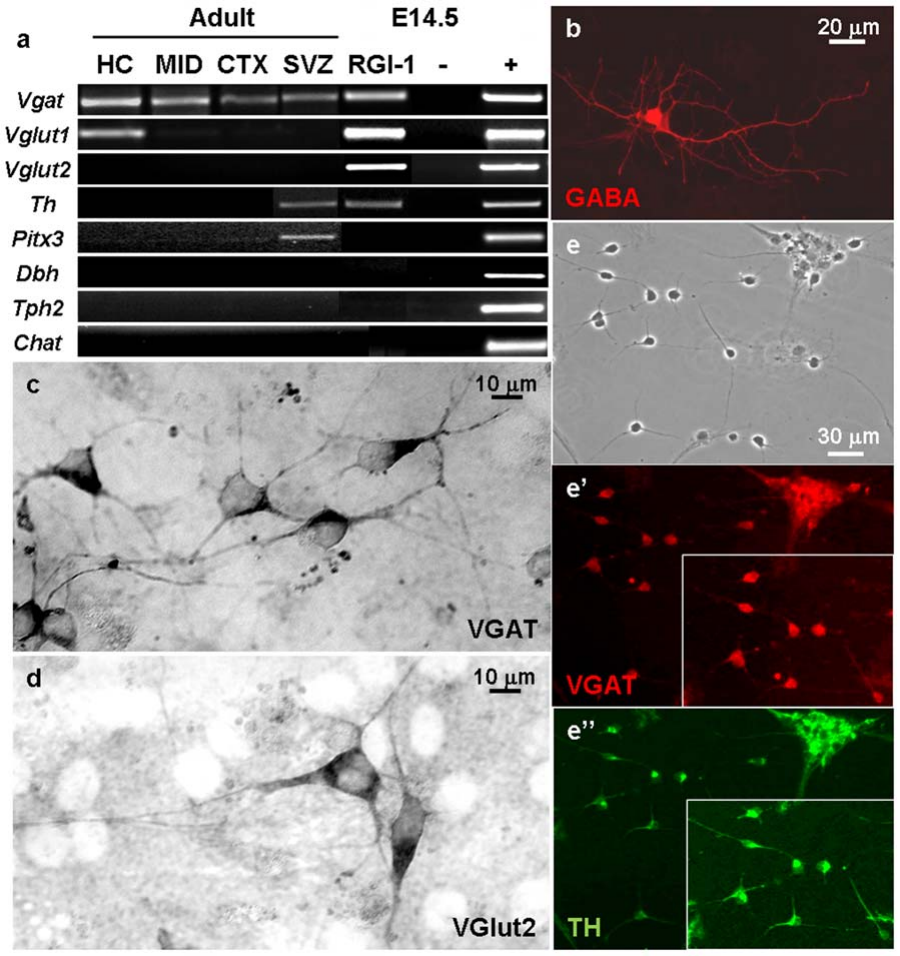
Different neuronal phenotypes developed from radial glia-like cells. Neurons with different neurotransmitter-phenotypes developed from cloned populations of RGl-cells. Genes indicating noradrenergic (*Dbh*), serotonergic (*Tph2*) and cholinergic (*Chat*) neurotransmitter phenotypes were not expressed in neuron-rich cultures of any RGl-cells regardless of fetal or adult origin (**a**). GABA- and VGAT-immunopositive GABAergic (**b**, **c** respectively) and VGlut2-immunopositive glutamatergic (**d**) neurons developed in embryo-derived clones upon EGF-withdrawal (cells from clone C4 are shown). All adult-derived clones generated GABAergic neurons. Hippocampus- derived HC_A cells produced *VGlut1*-expressing (**a**) neurons, and all SVZ-clones gave rise to tyrosine-hydroxylase (TH)-positive (**e, e’**) but *dbh*-negative (**a**), neurons (**e**, **e’**: neurons from SVZ_M clone are shown). The pictures were taken on the 11^th^ day after EGF-withdrawal.

RT-PCR analyses demonstrated that marker genes characteristic to GABAergic, glutamatergic and dopaminergic neurons were upregulated in embryo-derived RGl cells upon EGF-withdrawal (Figure 7a). Genes indicating the development of noradrenergic [dopamine-β-hydroxylase (*Dbh*)], serotonergic (*Tph2*) or cholinergic [choline-acetyl-transferase (*Chat*)] neurotransmitter phenotypes, however, were not activated (Figure 7a). Among E14.5-derived neurons, immunocytochemical staining revealed GABAergic (Figure 7b, c) and glutamatergic (Figure 7d) cells, approximately at the same frequency, but monoaminergic or cholinergic neuronal phenotypes were not found.

All investigated clones, regardless of origin, expressed the vesicular GABA transporter (vGAT) after the initiation of neuronal differentiation (Figure 7a). In differentiated cultures of embryo-derived RGl cells, both vGlut1 and vGlut2 neuron-specific glutamate transporters were transcribed. In adult RGl-derived neuronal cultures, on the other hand, vGlut1 was expressed only by the hippocampal (HC_A) clone and vGlut2 was not transcribed in any of the adult-derived neuronal cultures (Figure 7a).

Tyrosine-hydroxylase (TH)-immunopositive neurons (Figure 7e, e”.) and TH mRNAs (Figure 7a.) were found in neuronally differentiated cultures of fetal forebrain and adult SVZ-derived clones, but not in others. The same neurons also expressed GABAergic markers (GABA and vGAT; Figure 7e’). The lack of DBH mRNA indicated that SVZ clones give rise to dopamine-producing neurons. Formation of serotonergic and cholinergic phenotypes could not be demonstrated either at the mRNA (Tph2 and Chat, respectively) level or by immunocytochemical methods. Phenotypic markers expressed by different RG-clones are summarized in Table 2.

**Table 2:** Characteristics of differentiated cultures of cloned RG cells.

Clone	Embryonic (E14.5)			Adult (P 50-75)						
	A2	C4	RGl-1	HC_A	CTX_H	MES_D	SVZ_I	SVZ_K	SVZ_T	SVZ_M
neurons (IIIβ-tubulin immunreactive cells)	+(!)	+(!)	+(!)	+	+	+(!)	+	+	+	+
astrocytes (GFAP immunreactive cells)	+	+	+	+	+	+	+	+	+	+
oligodendrocyte precursors (O4- immunreactive cells)	+ (!)	+	ni	+ (!)	+(!)	+	+	+	+	+
	Gene expression									
*vgat*	ni	ni	+	+	+	+	+	+	+	+
*vglut1*	ni	ni	+	+	-	-	-	ni	ni	-
*vglut2*	ni	ni	+	-	-	-	-	ni	ni	-
*pitx3*	ni	ni	-	-	-	-	+	+	+	+
*tph2*	ni	ni	-	-	-	-	-	-	-	-
*dbh*	-	-	-	-	-	-	-	-	-	-
*chat*	ni	ni	-	-	-	-	-	-	-	-
*th*	ni	ni	+	-	-	-	+	+	+	+

ni: non investigated; (!): large frequency.

## Discussion

In early phases of neural tissue genesis, radial glial cells represent the major cell type in the neural tube and comprise the neurogenic population of the central nervous system [2]. Neural stem cells in the adult SVZ are derivatives of embryonic radial glial cells [22], and radial glia-like cells seem to persist in the adult brain [23]. In order to characterize and compare the characteristics of different neural stem/progenitor populations, purified fractions of such cells are required in a state preserving at least some native features.

Selective adhesion of non-differentiated cells to AK-cyclo[RGDfC]-coated surfaces, and the repulsion of neurons from the same surface [9] provided a simple and efficient way to enrich neural stem/progenitor cells in primary adherent cultures. As it was expected, non-differentiated neural stem/progenitor cells were selectively collected on AK-cyclo[RGDfC]-coated surfaces from suspensions of fetal forebrain cells containing mature glial cells at low frequency [24]. Serum-free conditions, which are known to hinder the survival and growth of mature glial cells promoted the adhesion-based selection of stem/progenitor cells also from adult brain tissues. Beside simple isolation, adhesion to AK-cyclo[RGDfC] allowed propagating fetal and adult brain-derived stem/progenitor cells in serum- and xenomaterial-free conditions.

Despite the reproducibility of the procedure, the molecular mechanisms behind the observed adhesive preferences are not understood. Cyclic RGD-containing pentapeptides were reported to be potent ligands of α_v_β_3_/α_v_β_5_ integrins [25]. α_v,_ β_3_ and β_5_ integrin mRNAs are present in RGl cells, but together with other integrin subunit mRNAs (data not shown). Moreover, we do not know yet, whether the binding preferences to selected integrin-complexes were preserved after conjugating the cyclic RGDfC motif into the brush-like peptide backbone [9]. Collaborative biochemical and cell biological studies are in progress to clarify some molecular mechanisms behind the preferential adhesive features of the AK-cyclo[RGDfC] polypeptide toward non-differentiated cells.

Another open question is the apparently reduced growth factor demand of the cells growing on AK-cyclo[RGDfC] coated surfaces. In serum-free medium supplemented only with EGF (beside the B27 commercial supplement), AK-cyclo[RGDfC]-adherent cells proliferated without differentiation, for several weeks. *In vivo,* the importance of neuregulins, members of the EGF family of ligands acting on ErbB-type receptors [26], have been well documented in the formation and persistence of the radial glial phenotype [27], [28], [29]. Accordingly, EGF was shown to be indispensable for *in vitro* survival of radial glia-like cells [30]. On conventional adhesive surfaces, however, initiation of adherent cultures of NS cells required at least bFGF besides EGF [31]. The reduced demand for growth factors on AK-cyclo[RGDfC]-coated surfaces might be reasoned by a massive stimulation of integrin signal-pathways by the cyclic RGD moieties spaced by regular nano-scale distances. Signalling through integrin receptors are known to crosstalk with multiple growth factor signalling pathways, and affect several basic cellular functions including survival, proliferation and differentiation [7], [8]. Ongoing studies will decide, whether the integrin-stimulation, alone, could provoke intracellular responses, sufficient to replace some growth factor effects, or, alternatively, it might initiate the autocrine production of some of the required factors.

he special adhesive features of AK-cyclo[RGDfC]-coated surfaces allowed isolating and amplifying neural stem/progenitor cell populations from fetal forebrain and from different regions of the adult mouse brain. The data indicate that AK-cyclo[RGDfC]-adherent, nestin and RC2 immunoreactive RGl cells share a number of features regardless of regional and developmental origin.

- RGl cells *preserve developmental capability to give rise to neurons, astrocytes and also to oligodendrocytes* as it was seen in cultures of pheno- and genotypically identical stem/progenitor cells, regardless of fetal or adult, ventral forebrain, hippocampal or midbrain origin. The finding does not rule out the existence of more restricted progenitors, but gives evidence on the preservation of multipotential stem cell populations in different regions of the adult mouse brain.
- *Large delayed rectifying potassium currents associated with passive conductance seems to be a unique feature of RGl cells*. Electrophysiological characterization showed that these cells display several bioelectric features characterizing also early embryonic neuroectodermal stem cells [32] and fetal subventricular zone progenitors [33]. Recordings from neural stem/progenitor cells in situ or from brain slices might confirm, whether this current profile may serve as a physiological marker for the identification of radial glia like progenitors. The passive conductance recorded from these cells indicates rapid redistribution of ions in a gap junction-coupled, enlarged cytoplasmic volume [32].
- *Neuronal cell fate commitment in RGl cells could not be induced by all-trans retinoic acid* (RA). In a number of self-renewing cells including embryoid body forming ES cells [34], embryonic carcinoma cells [35] or cells isolated from the early embryonic (E9 mouse) neuroectoderm [10], neural cell fate commitment can be provoked by retinoic acid treatment. In previous studies on embryonic (E9) neuroectoderm-derived NE-4C stem cells, we showed that after an initial cell fate commitment, retinoic acid does not promote neuronal differentiation [36]. The observations suggest that RGl cells represent a more advanced state of neural cell fate commitment in comparison to early embryonic neuroectodermal progenitors. Underlying the assumption, RGl cells – even embryo-derived ones - gave rise readily to astrocytes, in a much shorter term than the investigated early embryonic stem cells [10], [37].
- In vitro induced neuronal differentiation could result in the *formation of neurons with diverse* – GABAergic, glutamatergic and in some clones also catecholamine-producing – *neurotransmitter phenotypes* from identical (one-cell-derived) RGl cells. While NS cells were reported to give rise only to GABAergic neurons [31], embryo- and hippocampus-derived RGl cells on AK-cyclo[RGDfC] surfaces produced glutamatergic neurons at high frequency. Activation of integrin receptors [38], as well as the addition of bFGF [39] can alter the fate of cultured neural progenitors. Cortical progenitors were shown to develop into GABAergic rather than glutamatergic neurons in response to bFGF [39]. In our protocol, the ready formation of glutamatergic neurons might be explained by the lack of bFGF supplementation. The data however clearly show the flexibility of neural stem/progenitor cells, even those residing in the adult brain.
- In vitro propagated *RGl clones expressed “positional” genes which, during in vivo development, are not transcribed in overlapping territories*. In contrast to the preservation of some regional differences in cellular phenotypes, the expression pattern of “positional genes” did not necessarily reflect the regional origin of the clones. Several genes (as Ngn2) were expressed by cells cloned from “relevant” position and/or did not appear “ectopically”: *Ngn2* was expressed by embryonic dorsal forebrain-derived clone, but not by the ventral-derived one; *Hoxb2* was not expressed by any of the clones and Nkx2.1 was not expressed by clones with dorsal origin. A number of position-indicating genes including *Gbx2, Emx2, Dlx2, Otx2, En1, Mash1*, however, were expressed by all clones regardless of origin. In contrast to the conclusion of a recent paper [40], our data indicate, that neural stem/progenitor cells do not necessarily preserve the region-specific expression profiles of each investigated genes, if isolated from their native environment and propagated in vitro. We have to admit however, that investigating clones restricts the studies to populations derived from a few selected founder-cells and can not provide data on the regional commitment of the whole variety of neural stem/progenitor cells. Some regional features, however, were preserved in the investigated clones. From fetal clones, *Ngn2* was expressed by the dorsal forebrain-derived clone, but not by the ventral-derived one; oligodendrocyte production was higher in the ventral-derived one in comparison to clones of dorsal origin. Even more striking region-dependent differences were found in the expression of neurotransmitter phenotype indicating genes. In accordance with the in vivo pattern [41], *Vglut1* was indeed expressed exclusively by neuronal progenies of hippocampus-derived RGl cells. Similarly, corresponding to in vivo data, [42], [43], only SVZ-derived clones gave rise to tyrosine-hydroxylase immunoreactive neurons. These and previous data [44] indicate that isolated neural stem/progenitor cells while displaying important flexibility, restrain some age- and region-specific determination. For the time being, the right markers are missing for recognizing the level of regional or neuronal sub-type determination.

The isolation of RGl clones from non-neurogenic adult cortical and midbrain regions showed that non-differentiated progenitors reside in the adult brain parenchyma. The randomly selected clones could produce both glial cells and neurons. The finding raises questions concerning the developmental stage of scattered progenitors and also the uncertainties about a low-rate but permanent neurogenesis in the mammalian brain parenchyma (reviewed by [5]).

In our hope, the simplified adhesion-based isolation and serum-free maintenance of radial glia-like cells can accelerate the collection and characterization of RGl cells from well-identified territories of both neurogenic and ”non-nerugenic” brain zones.

## Materials and Methods

### Preparation of adhesive coatings

Aliquots of AK-cyclo[RGDfC] stock solution (1 mg/ml; in distilled water) were stored at -20°C. Peptide solutions were diluted to 10 µg/ml with distilled water just before use and polystyrene or glass culture surfaces were covered with a volume containing 0.25 µg peptide for each cm^2^ of the surface (e.g. 50 µl, 500 µl, 1 ml and 2 ml for 96-, 24-well plates, 35 mm and 60 mm tissue culture dishes, respectively). The solutions were left on surfaces for 30 min at room temperature, and then aspirated. The surfaces were let to dry under sterile air stream. The estimated peptide density was about 0.25 µg/cm^2^, pretending that the vast majority of these “sticky” peptides was absorbed on the surface. For control, surfaces were coated with poly-L-lysine (PLL; Sigma) by using 10 µg/ml PLL in distilled water according to the above protocol. After drying, coated surfaces could be stored for up to 4 months at 4°C. The surfaces were rinsed with serum-free tissue culture medium prior seeding the cells.

### Preparation of primary cell suspensions

Animal experimentation licensed by local authorities (license No.: 22.1/3894/003/2009) was carried out by paying special attention to the ethical rules of animal experimentation in accord with the European Community Council Directives (86/609/EEC and 2010/63/EU).

### Embryo-derived suspensions

Timed pregnant wild-type CD1, or hGFAP-GFP [17] and CD1/EGFP [19] transgenic mice were sacrificed by over-dose injection of ketamin/xylazin aenesthetics on day 14-16 post-conception. Telencephali of 10-25 embryos were aseptically removed and placed into sterile PBS. The meninges were removed under dissecting microscope (Zeiss) and pallial or subpallial tissue parts were cut into small (∼ 1 mm^3^) pieces. Tissue pieces were mechanically disintegrated by triturating with a fire-polish transfer pipette in DMEM (Sigma). The suspension was filtered through a nylon mesh with pore diameter of 45 µm and the cell-yield in the single cell containing filtrate was determined by counting in haemocytometer.

### Adult-derived suspensions

Adult CD1 mice were sacrificed by over-dose injection of ketamin/xylazin aenesthetics. The brains were aseptically transferred into PBS. The meninges were removed and the desired brain areas were dissected under dissecting microscope (Zeiss Jena, Germany). The collected tissues were cut into small pieces and enzymatically dissociated using the Neural Tissue Dissociation Kit (Miltenyi Biotec) according to the manufacturer's instructions. Cell suspensions were prepared from the dorso-lateral and ventro-lateral linings of the forebrain ventricles, from the hippocampus, from the parietal cortex and from the dorso-lateral parts of superior colliculi.

### Cultures of radial glia-like (RGl) cells

Cell suspensions were centrifuged (120 g; 10 min) and quickly re-suspended (to avoid aggregation) in basal RGl-medium composed by DMEM/F12 (1/1) (Sigma) and 1% B27 supplement (Gibco, Invitrogen). 2×10^5^ cells/cm^2^ were plated onto AK-cyclo[RGDfC]- or PLL-coated dishes. After seeding, the basal RGl medium was supplemented with 20 ng/ml EGF (Peprotech) (complete RGl-medium).

The medium of *embryo-derived cultures* was changed every second day. Before adding fresh medium, the cultures were rinsed with sterile PBS to wash off weakly adhering cells. *Adult-derived cultures* were not washed and only the half of the medium was changed every second day, during the first week.

At the end of the first week, when fetal cultures reached confluency and colonies developed in the adult-derived cultures, the cells were harvested by rinsing with trypsine solution (0.05% trypsine, 1 mM EDTA in PBS; 1 min at room temperature) and subsequent washing off with basal RGl medium. Cell suspensions were split, and reseeded into fresh AK-cyclo[RGDfC]-coated dishes at densities of 10^5^ cells/cm^2^. After the first passage, the cultures could be subcultivated on every second or third day. After 3-4 passages, cultures comprised virtually homogeneous populations of radial glia-like cells.

### Establishment of one-cell derived clones

After 4 passages, cultures of were harvested by trypsinization and diluted to achieve spare, single cell attachment in AK-cyclo[RGDfC]-coated 90 mm or 60 mm dishes. 4-6 hours after plating, attached single cells were isolated by cloning rings. Colonies developed inside the individual rings were regarded as one-cell-derived clones.

### Viability assays

The viability of RGl cell cultures was determined by MTT- [3-(4,5-Dimethylthiazol-2-yl)-2,5-Diphenyltetrazolium Bromide; Sigma] reduction assay [45] in the presence or absence of EGF and the EGF receptor inhibitor, AG1478 (0.25 µM; Calbiochem). For each data point, results of 4 to 8 identically treated sister cultures were averaged and standard deviations were calculated.

### Differentiation of radial glia-like cells

For large-scale neuron-production, EGF was withdrawn from the media of confluent cultures of RGl cells. Neuronal differentiation was monitored by phase contrast microscopy of living cultures and by immunocytochemistry after 6–12 days.

For astrocytic differentiation, medium was supplemented with 5% Fetal Calf Serum (FCS; Sigma). The presence of GFAP-immunpositive cells was checked from the third day.

The development of oligodendrocytes was reached by a 4+4-day protocol, according to Glaser et al., 2007. Briefly, the cells were cultured in basal RGl-medium supplemented with FGF2 (10ng/ml; Peprotech), PDGF (10ng/ml; Sigma) and forskolin (10 µM; Sigma), for 4 days. The medium was then replaced with DMEM/F12 (1/1) containing 3,3,5-triiodothyronine (T3; 30 ng/ml; Sigma) and ascorbic acid (200 µM; Sigma) as only supplements. At the end of the 8^th^ day, the presence of oligodendrocytes was checked by immunocytochemical staining.

### RT-PCR analyses

Radial glia-like cells were lysed by addition of Tri Reagent (Sigma) according to the manufacturer's recommendation. Total RNA fraction was then isolated using organic/inorganic extraction by the standard procedures. DNA contamination was eliminated by DNase-I (Fermentas) treatment. The isolated RNA was suspended in RNase/DNase free water at a concentration of 1 µg/µl and stored at −70°C. Reverse transcription (RT) reactions were undertaken from 1.5 µg total RNA using First strand cDNA synthesis Kit (Fermentas) at 42°C. The quantity and the potential genomic DNA contamination of the cDNA product was determined by PCR [Hotstart Taq PCR Kit (Qiagen)] using primers recognizing both cDNA (248bp) and genomic DNA (1086bp) sequences of the house keeping hypoxanthine guanine phosphoribosyl transferase (*Hprt*) gene. Genomic DNA-free cDNA samples were diluted to equal cDNA content verified by *Hprt* amplification. The primer pairs used for PCR analyses are shown in Table 3. PCR products were run in agarose gels containing 0.5% ethidium bromide, and were visualized by UV trans-illumination.

**Table 3:** PCR primer sequences.

Gene	Sequences
*Pax6*	Forward: 5′-ACGAAAGAGAGGATGCCTC-3′
	Reverse: 5′-CCCAAGCAAAGATGGAAG-3′
*Sox2*	Forward: 5′-GCCCTGCAGTACAACTCCAT-3′
	Reverse: 5′-ACCCCTCCCAATTCTCTTGT-3′
*Blbp*	Forward: 5′-ACCCGAGTTCCTCCAGTTC-3′
	Reverse: 5′-CAAAAGCAAGTTCCCATTCA-3′
*Glast*	Forward: 5′-TGGGTTTTCATTGGAGGGTTG-3′
	Reverse: 5′-CAGTACGTTGGTGGTGGTTCG-3′
*Gfap*	Forward: 5′-GACTATCGCCGCCAACTGC-3′
	Reverse: 5′-CGTCCTTGTGCTCCTGCTTC-3′
*Math2*	Forward: 5′-TGAGAATGGCTTGTCCAGAAGG-3′
	Reverse: 5′-TGGTAGGGTGGGTAGAATGTGG-3′
*Oct4*	Forward: 5′-GGCGTTCTCTTTGGAAAGGTGTTC-3′
	Reverse: 5′-CTCGAACCACATCCTTCTCT-3′
*Nanog*	Forward: 5′-GCGCATTTTAGCACCCCACA-3′
	Reverse: 5′-GTTCTAAGTCCTAGGTTTGC-3′
*Mash1*	Forward: 5′-GTTGGTCAACCTGGGTTTTG-3′
	Reverse: 5′-GTGATTCGGGCTTAGGTTCA-3′
*Ngn2*	Forward: 5′-AAGAGGACTATGGCGTGTGG-3′
	Reverse: 5′-ATGAAGCAATCCTCCCTCCT-3′
*Emx2*	Forward: 5′-GTCCCAGCTTTTAAGCTAGA-3′
	Reverse: 5′-CTTTTGCCTTTTGAATTTCGTTC-3′
*Nkx2.1*	Forward: 5′-CCGCCTTACCAGGACACCA-3′
	Reverse: 5′-CCGCCCATGCCACTCATAT-3′
*Hox2b*	Forward: 5′-GCTGGAGAAGGAGTTCCACT-3′
	Reverse: 5′-TAGGGAAACTGCAAGTCGAT-3′
*Dlx2*	Forward: 5′-CAGGGTCCTTGGTCTCTTCA-3′
	Reverse: 5′-CTGCTGAGGTCACTGCTACG-3′
*Vgat*	Forward: 5′-ACGAGGAGAACGAAGACGG-3′
	Reverse: 5′-ACGATGATGCCAATGGAGAT-3′
*Vglut1*	Forward: 5′-TTGAGGGCTTTATTTGGAGGG-3′
	Reverse: 5′-CGTACACCAGAGCGTTTATTGG-3′
*Vglut2*	Forward: 5′-TGGAAAATCCCTCGGACAGA-3′
	Reverse: 5′-TAGACGGGCATGGATGTGAA-3′
*Th*	Forward: 5′-GCGGCAGAGTCTCATCG-3′
	Reverse: 5′-CCTCCTTCCAGGTAGCAA-3′
*Pitx3*	Forward: 5′-GTTATCGGACGCAGGCA-3′
	Reverse: 5′-TGAAGGCGAACGGGAAG-3′
*Dbh*	Forward: 5′-CATTACCACAACCCACGGAA-3′
	Reverse: 5′-AGTCATACAGAGCCTTGAGCATA-3′
*Tph2*	Forward: 5′-TCTCCAAACTCTACCCGACTC-3′
	Reverse: 5′-AACCCTGCTCCATACGC-3′
*Chat*	Forward: 5′-GAAAGATAGTCAAAATGGCGTCC-3′
	Reverse: 5′-CCAGGCATACCAGGCAGAT-3′

### Immunocytochemistry

For immunochemical staining, the cells were fixed with 4% paraformaldehyde (Taab; w/v in PBS) for 20 minutes at room temperature, permeabilized with 0.1% Triton-X 100. Non-specific binding was blocked by incubating with 2% bovine serum albumin (in PBS) for 1 hour. Primary antibodies recognizing RC2 (DSHB, 1/500), MAP-2 (Chemicon, 1/100), nestin (Chemicon, 1/1000), GABA (Sigma, 1/1000), VGAT (Synaptic Systems, 1/500), VGlut2 (Chemicon, 1/200), tyrosin-hydroxylase (TH) (Chemicon; 1/500), GFAP (Sigma, 1/1000), O4 (Chemicon, 1/10) or βIII-tubulin (Exbio, 1/1000) were used at 4°C, overnight. For fluorescent visualization, anti-mouse-Alexa-594 (Invitrogen, 1/1000) and anti-rabbit-Alexa-488 (Invitrogen, 1/1000) secondary antibodies were used for one hour at room temperature. In case of DAB- (3-3′-diamino-bensidine) staining, cultures were incubated with biotin-conjugated secondary (anti-rabbit or anti-mouse) immunglobulins (Vector, 1/1000) for one hour. For visualization, Vectastain ABC kit (Vector), and then DAB (0.55 mg/ml) with 0.3% H_2_O_2_ were used. Stained preparations were investigated with a Zeiss Axiovert 200M light/fluorescent microscope.

### Counting of metaphase chromosomes

Cells were hypotonized with 0.56% KCl and with distilled water for 10+10 minutes and fixed with methanol/acetic acid (3/1) on ice, for 20 min. Fixed cells were dropped onto glass slides, and chromosomes were counted under phase contrast microscope (Nikon TS100).

### Patch-clamp recordings and electrophysiological measurements

Transmembrane currents were recorded by patch-clamp technique in whole-cell configuration. Recording pipettes had a tip resistance of 3-5 MΩ. Electrodes were filled with a solution containing 130 mM KCl, 0.5 mM CaCl_2_, 2 mM MgCl_2_, 5 mM EGTA, 10 mM HEPES; pH = 7.2. The extracellular solution contained 145 mM NaCl, 3 mM KCl, 2 mM CaCl_2_, 1 mM MgCl_2_, 10 mM D-Glucose, 10 mM HEPES; osmolality 300 mmol/kg. Current signals were amplified with MultiClamp700B amplifier (Axon Instruments), lowpass-filtered at 4 kHz and digitized at 5 kHz by CED micro1401 interface (Cambridge Electronic Design). Data acquisition, storage and analysis were performed with Strathclyde Electrophysiology Software Whole Cell Program (by John Dempster).

Current patterns were obtained by clamping the cell membrane from a holding potential of -70 mV to values ranging from −160 mV to +20 mV, at 10 mV intervals. Pulse duration was 50 ms. Amplitudes of K_DR_ were measured at +40 mV, at 40 ms. Na^+^ current amplitudes were measured at the peak value. All values are expressed as means ± standard errors of means (S.E.M.).
